# A Monte Carlo study on electron and neutron contamination caused by the presence of hip prosthesis in photon mode of a 15 MV Siemens PRIMUS linac

**DOI:** 10.1120/jacmp.v14i5.4253

**Published:** 2013-09-06

**Authors:** Mohammad Taghi Bahreyni Toossi, Marziyeh Behmadi, Mahdi Ghorbani, Hamid Gholamhosseinian

**Affiliations:** ^1^ Medical Physics Research Center Medical Physics Department, Faculty of Medicine Mashhad University of Medical Sciences Mashhad Iran; ^2^ Medical Physics Department, Faculty of Medicine Mashhad University of Medical Sciences Mashhad Iran

**Keywords:** electron contamination, hip prosthesis, Monte Carlo, neutron contamination, Siemens PRIMUS linac

## Abstract

Several investigators have pointed out that electron and neutron contamination from high‐energy photon beams are clinically important. The aim of this study is to assess electron and neutron contamination production by various prostheses in a high‐energy photon beam of a medical linac. A 15 MV Siemens PRIMUS linac was simulated by MCNPX Monte Carlo (MC) code and the results of percentage depth dose (PDD) and dose profile values were compared with the measured data. Electron and neutron contaminations were calculated on the beam's central axis for Co‐Cr‐Mo, stainless steel, Ti‐alloy, and Ti hip prostheses through MC simulations. Dose increase factor (DIF) was calculated as the ratio of electron (neutron) dose at a point for $10\times 10\;{\text{cm}}^{2}$ field size in presence of prosthesis to that at the same point in absence of prosthesis. DIF was estimated at different depths in a water phantom. Our MC‐calculated PDD and dose profile data are in good agreement with the corresponding measured values. Maximum dose increase factor for electron contamination for Co‐Cr‐Mo, stainless steel, Ti‐alloy, and Ti prostheses were equal to 1.18, 1.16, 1.16, and 1.14, respectively. The corresponding values for neutron contamination were respectively equal to: 184.55, 137.33, 40.66, and 43.17. Titanium‐based prostheses are recommended for the orthopedic practice of hip junction replacement. When treatment planning for a patient with hip prosthesis is performed for a high‐energy photon beam, attempt should be made to ensure that the prosthesis is not exposed to primary photons.

PACS numbers: 87.56.bd, 87.55.kh, 87.55.Gh

## I. INTRODUCTION

Currently, the number of patients with hip prosthesis undergoing pelvic irradiation in radiotherapy is growing.^(1)^ According to the report of Task Group No 63 (TG‐63) of American Association of Physicists in Medicine (AAPM), between 1% and 4% of radiotherapy patients have prosthetic devices which could affect the predetermined value of the dose that they would receive. This Task Group was formed to identify the problems caused by the presence of high‐Z prosthesis material in radiation therapy in the megavoltage photon energy range.^(1)^

Due to the importance of electron and neutron contamination in radiation therapy, several researchers have performed various studies in this field.^2^, ^3^, ^4^, ^5^, ^6^, ^7^, ^8^, ^9^, ^10^ Electrons produced in the target, flattening filter, and primary collimator may have a chance to reach the patient's body surface, which in tum would increase the skin dose. When a cancer patient is undergoing irradiation, various severe skin reactions may occur. Some of the early effects are erythema and desquamation. Occasionally, telangiectasia is a late effect. Subcutaneous tissue fibrosis may occur in high dose levels. The treatment aim is not to irradiate the skin. thus the amount of skin dose must be taken into account when establishing the treatment criteria.^(6)^

In photonuclear reaction. a photon collides with a nucleus. then one or more neutrons are ejected from the nucleus. This interaction is more probable when the energy of the incident photon is higher than the threshold energy required for the reaction. This threshold energy depends on the atomic number of the target: while for elements of high atomic number it is around 8 MeV, for low atomic numbers the threshold energy is higher: 16 MeV for oxygen and 18 MeV for carbon.^(11)^ Linacs with photon energies greater than 8 MeV include an undesired additional dose to patients due to neutron contamination.^(12)^ Neutron production is mainly due to interactions of photons and electrons of high energies with the high‐Z materials used in the construction of linac head or every other high‐Z component containing a high‐Z material in the beam pathway (e.g., target, collimator, and wedge).^(13)^ Due to their higher RBE, photoneutrons are a source of unwanted out‐of‐field exposure to patients. Several authors have pointed out the possibility of associated risks of secondary cancers following radiation therapy which are related to neutron contamination.^(^ ^8^ ^,^ ^14^ ^,^ ^15^ ^)^ Various studies have been performed to assess the impact of a hip prosthesis on the distribution and attenuation of photon dose in radiation therapy.^(^ ^1^, ^16^ ^‐^ ^25^ ^)^

AAPM TG‐63 has identified all problems that are caused by the presence of metal implants in radiotherapy. This report has also paid attention to the neutron production when pelvic tumors are treated with photons of higher energy than 10 MeV. However, the report focuses only on additional dose from thermal neutron capture processes in the metal prostheses. In the AAPM report, it is noted that in a linac, extra neutron‐induced photon dose is less than 0.5% of the photon dose at 1 cm from the prosthesis; therefore, it is assumed to be clinically negligible.^(^ ^1^ ^,^ ^26^ ^)^ The report is more focused on the effects of prosthesis on photon dose distribution rather than the production of neutron contamination by metal prostheses in the beam pathway.^(26)^

Few researchers have paid attention particularly to neutron contamination produced by various materials present in the hip prosthesis. Schneider et al.^(15)^ have measured the neutron dose originated from a prosthesis material when radiation therapy was performed with photon and proton beams. Their measurements were limited only to aTi‐alloy prosthesis. They did not find any influence by high‐Z prosthesis material on the neutron dose production by both photon and proton interactions. Becker et al.^(9)^ have investigated the neutron contamination in 15 MV photon mode of a Siemens Primus linac, and then have estimated the impact of a titanium hip prosthesis on neutron contamination. Their study was based on Monte Carlo simulation of the linac's head and they have found that total neutron dose was increased only to 12% (or 4 μGy) per 100 monitor unit (MU) of photon dose when the prosthesis was present in a phantom.

Most hip prostheses are made of cobalt‐chrome alloys, because they are considered to have the best combination of corrosion resistance, resistance to fatigue, and mechanical strength. However, both titanium and stainless steel hip prostheses are also available.^(^ ^24^ ^,^ ^25^ ^,^ ^27^ ^)^ Various metallic materials in combination with polymeric plastics are used in manufacturing artificial hips. These implant materials are chosen to provide the necessary strength and resistance. They are also biostable and compatible with body tissues. The vast majority of artificial hips in clinical use are made of iron‐cobalt, or titanium‐based alloys.^28^, ^29^ Different elements in prostheses could affect the quantity of electron contamination. To the best of our knowledge, the electron contamination produced by hip prosthesis present in a patient or a tissue equivalent phantom has not been examined to date. Furthermore, different prostheses could produce different amounts of neutron contamination due to varying photonuclear cross section of their consisting elements.

In the present study, a 15 MV Siemens PRIMUS linac was simulated to estimate electron and neutron contaminations produced by various hip prostheses. For this purpose, four hip prostheses with various materials which are more frequently used in orthopedic procedures were simulated by MCNPX Monte Carlo code, and their impacts on electron and neutron contamination were estimated and compared.

## II. MATERIALS AND METHODS

### A. Monte Carlo simulations and verifications

A MCNPX (version 2.6.0) radiation transport Monte Carlo code was used in this study to simulate a Siemens PRIMUS medical linear accelerator (Siemens AG, Erlangen, Germany). This code allows development of a detailed three‐dimensional (3D) model of a linear accelerator treatment head and dose calculation in complex geometries and materials.^(30)^ The code has been used in many studies in medical linac head simulations, calculation of dose distributions in treatment planning, as well as other aspects of medical radiation dosimetry.^(31)^

**Figure 1:**
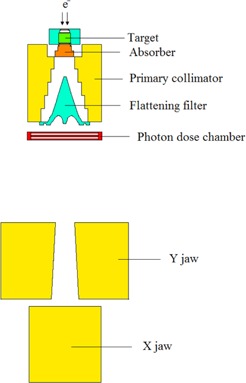
A schematic geometry of the head of Siemens PRIMUS linac at 15 MV photon mode used in this study.

In this study we have simulated 15 MV photon mode of a Siemens PRIMUS linac based on the geometric information provided by the manufacturer. A schematic diagram illustrating the components of the Siemens PRIMUS linac head (15 MV photon energy) is presented in Fig. 1.^(8)^ In our model, we have incorporated the main components in the beam path. The main components of the linac's head for 15 MV photon mode are: target (9 layers), primary collimator (6 layers), absorber (4 layers), flattening filter (16 layers), photon dose chamber (5 layers), as well as Y and X jaws. The target is composed of stainless steel (SST), graphite, gold, water, and air. Primary collimator is made of tungsten which is placed under the target. The absorber is made of aluminum with 1.27 cm height and the flattening filter layers are composed of SST with 4.877 cm height from base to the top. The photon chamber layers are composed of alumina $\left({\text{Al}}_{2}O_{3}\right)$ and air with total thickness of 0.826 cm from top to bottom. The Y and X jaws, which are made of tungsten, were defined under the photon chamber in our simulations.

To perform dose calculations for a water phantom, two input files were defined and run. In the first input file, a horizontal plane, was defined above the Y jaws at a distance of 19.7 cm from the electron source. The input file was run for $2\;\times \;10^{9}$ source particle histories. The energy cutoff for electron and photon was defined as 0.2 and 0.01, respectively. Due to this run, a number of $640\;\times \;10_{6}$ particles (photons and electrons) were scored on the horizontal plane. For this purpose, surface source write (SSW) card, which is an option in MCNPX code, was utilized. In the second input file, particles from the horizontal plane were read by surface source read (SSR) card and then the absorbed dose values were scored in a predefined water phantom of $30\;\times \;30\;\times \;30\;{\text{cm}}^{3}$ in dimensions. The water phantom was positioned under the treatment head at a source to surface distance (SSD) of 100 cm. The dose values were then utilized to calculate percentage depth dose (PDD) and dose profile data. To choose the optimum energy to provide the best agreement with the measured values of PDD and dose profiles, secondary collimators were set to create a field size of $10\times 10\;{\text{cm}}^{2}$ on the phantom surface. The optimum input electron energy spectrum colliding on the target was selected among the ± 0.5 MeV energy range relative to the manufacturer‐provided energy spectrum. The selection of the optimum energy was based on agreement of build‐up depth for the $10\times 10\;{\text{cm}}^{2}$ field size from Monte Carlo simulations and the experimental measurements. This optimum energy then was employed in the next steps for PDD and dose profile calculations for other field sizes, as well as electron and neutron contamination calculations. The agreement between the simulated and measured data was also examined by implementing gamma function.

Depth dose values and beam profiles acquired from simulations of various field sizes and depths were compared with analogous values obtained by measurements. To accomplish depth dose calculation for various depths of the water phantom, a cylinder was defined with 1 cm in radius (for $6\times 6\;{\text{cm}}^{2}$ field size) and 1.5 cm (for 10 × 10 and $18\times 18\;{\text{cm}}^{2}$ field sizes). The cylinder axis was assumed positioned on the beam central axis. The cylinder was then divided into tiny cells, 2 mm in height, named scoring cells, and the deposited energy in each scoring cell was determined by *F8 energy deposition tally, and was assigned as depth dose of the point of interest. The energy cutoffs for electrons and photons were set as to 0.5 and 0.01 MeV, respectively, in the simulations for PDD calculations. The same approach was followed to calculate beam profiles, expect that the main axes of scoring cylinders were perpendicular to central axis of the beam. Three cylinders were positioned at three depths of the water phantom: 5, 10, and 20 cm for each field size. For all field sizes the radius of the cylinder used for dose profile calculations was 2 mm. The energy cutoff for electrons and photons was defined as 0.5 and 0.01 MeV, respectively, in calculations of dose profile. All second input files were run for $6.48\;\times \;10^{8}$ particle histories. In PDD calculations, uncertainty in dose value in each depth was extracted from Monte Carlo output file. The value of uncertainty in depth of build‐up was also extracted from Monte Carlo output, and then the uncertainty in value of PDD was calculated as combined uncertainty from these two values by combining the uncertainties for division operation. The calculations were performed for various depths and field sizes and the maximum combined value was extracted. The same method was used for reporting the uncertainty of dose profiles, with difference that the midpoint in dose profile curve was the reference point in calculation of dose profile and the related combined uncertainty. The maximum combined Monte Carlo statistical uncertainty in PDD and dose profile calculations was equal to 3.49% and 5.13%, respectively.

To verify our simulation data, we have compared our Monte Carlo results with the corresponding measured values by using gamma function method. Measurements were performed on a Siemens PRIMUS medical linear accelerator with 15 MV nominal photon energy. Dosimetry was performed by using a Wellhofer‐Scanditronix dosimetry system (Wellhofer, Uppsala, Sweden) and a water phantom (RFA‐300; IBA Dosimetry GmbH, Schuarzenbruck, Germany) at the Reza Radiation Oncology Centre (Mashhad, Iran). This equipment is controlled by RFA‐plus software. The depth measurements were performed using a diode detector. The dimensions of the water phantom were 50 × 50 × 50 cm. In‐phantom measurements were performed according to Technical Reports series No. 398 reported by International Atomic Energy Agency (IAEA)^(32)^ and, based on this report, average uncertainty in the measurements was 1.61%.

Gamma function algorithm which was developed by Low et al.^(33)^ is a common method for comparing two dose distributions: one is defined as the reference information $\left(D_{r}(r)\right)$ and the other is queried for evaluation $\left(D_{c}(r)\right)$. The acceptance criteria are denoted by $\Delta D_{M}$ representative for the dose difference (DD) and $\Delta d_{M}$ representative for distance to agreement (DTA). These variables are defined in terms of percentage dose difference (%) and spatial tolerance (maximum allowable separation of isodose lines (mm)) between the two given dose distributions^34^, ^35^ The concept of a spatial tolerance or distance to agreement was previously introduced by several authors. While DTA alone when applied to dose distributions would be oversensitive in low‐dose gradient regions, DD is oversensitive to high‐dose gradient regions. As a consequence, several authors^(^ ^36^ ^,^ ^37^ ^)^ have combined DD and DTA variables to form a binary “gamma function” that yields value of 1 whenever both DD and DTA criteria are fulfilled, or 0 when either DD or DTA (or both) are not fulfilled at individual points in the evaluated dose distribution. The gamma function was later modified by Low and Dempsey^(38)^ to have continuous values, where gamma values between 0 and 1 are considered a pass and values exceeding 1 are considered to as fail. Gamma function criteria which are typically used are 3% for DD and 3 mm for DTA.^33^, ^39^, ^40^, ^41^ However other combinations have been reported to be used in the clinic.^(^ ^38^ ^,^ ^42^ ^,^ ^43^ ^)^ Most of the available gamma function software compare 2D dose distributions, but we needed a software capable of comparing 1D relative dose distributions with respect to our PDD and dose profile data. A special gamma function software has been prepared by DOSIsoft company (Cachan, France). The software (Gamma_index.exe) is working under Gnuplot software environment (version: 4.4 patch level 3, Geeknet Inc., Fairfax, VA). In the present study, in calculation for gamma function we have used dose difference and distance to agreement criteria equal to 3% and 3 mm, respectively. There are also two other studies on Monte Carlo simulation of linacs in photon mode with the criteria of 3% and 3 mm.^44^, ^45^

### B. Estimation of electron and neutron contaminations from hip prostheses

In the second stage of this work, we have simulated four prostheses, each assumed a cube with 4 × 4 × 4 cm in dimensions, made of various metallic compounds. In order to simulate pelvic prosthesis, the cubes were placed at depth of 12 cm in a water phantom. Various compositions including Co‐Cr‐Mo, stainless steel, Ti‐alloy, and titanium, which are in use more frequently in orthopedics, were chosen. The elemental compositions of the four hip prostheses are provided in Table 1.^(46)^

In order to assess how much prosthesis present in the phantom would affect the electron (neutron) contamination, the ratio of electron (neutron) doses in different points on beam's central axis with and without presence of prostheses was calculated. Dose increase factor (DIF) was calculated as the ratio of electron (neutron) dose at a point for $10\times 10\;{\text{cm}}^{2}$ field size in presence of prosthesis to that at the same point in absence of prosthesis. DIF values for total dose were also presented as tabulate data. Total dose in case of electron contamination was calculated as electron dose plus photon dose. Total dose for neutron contamination was calculated as neutron dose plus photon dose. In order to calculate electron contamination, we have used two types of input files: one for a free water phantom and one with presence of prosthesis on the beam's central axis in the water phantom. The electron dose values on the central axis of the beam were calculated for these types of input files. Each prosthesis material was studied by a separate input file. In these calculations, SSD was set to 100 cm and the field size was $10\times 10\;{\text{cm}}^{2}$. SSW and SSR cards have been used in the same way as were used for PDD and dose profile calculations. Photons and electrons were scored on a horizontal plane located at 19.7 cm from the source in the first run file. In the second run file, $35\;\times \;10^{7}$ particles were read from the plane and electron dose was scored at 1–29 cm depths in the water phantom. The scoring cells were cylinders with 1 cm radius and 2 mm height. The energy deposition was calculated in the scoring cells using *F8 tally. Since the photon doses were required for calculation of DIF for total dose (electron dose + photon dose), the photon doses were calculated using *F8 and F6 tallies in the same tally cells used for calculation of electron dose. In this case, *F8 tally was utilized for calculation of photon dose in depths ranging from 0.1–2.9 cm, and F6 tally in depths of 3.1–21 cm. The input files used for calculation of photon dose were run for about $1.41\;\times \;10^{8}$ particles and the Monte Carlo errors in the tally cells were less than 3.81%. Except for cutoff and cell importance, no other variance reduction method was used. Energy cutoff for both electrons and photons were set at 10 keV and cell importance was increased in the range of 100–600 in the water phantom with increasing depth.

**Table 1:** Elemental composition (percentage fraction by weight) and mass density (p) of the four hip prostheses materials used in this study

*Co‐Cr‐Mo Alloy* $\text{Ti}\;\rho \;=\;8.20\;(g/{\text{cm}}^{3})$		*Stainless Steel* $\rho \;=\;6.45\;(g/{\text{cm}}^{3})$		*Ti‐alloy* $\rho \;=\;4.48\;(g/{\text{cm}}^{3})$		*Ti* $\rho \;=\;4.506\;(g/{\text{cm}}^{3})$	
*Element*	${\text{WF}}^{a}\;(\%)$	*Element*	*WF (%)*	*Element*	*WF (%)*	*Element*	*WF (%)*
Co	61.90	Fe	62.72	Ti	89.17	Ti	100
Cr	28.00	Cr	21.00	Al	6.20		
Mo	6.00	Ni	9.00	V	4.00		
Mn	1.00	Mn	3.60	Fe	0.30		
Si	1.00	Mo	2.5	O	0.20		
Fe	1.00	Si	0.75	C	0.08		
Ni	0.75	N	0.43	N	0.05		
C	0.35						

a WF = weight fraction.

Neutron contamination was also calculated under similar conditions as for electrons. In other words, SSD, field size, scoring cells, and the types of input files and the implemented variance reduction methods were the same as described above for electron contamination simulations. However, there were some differences in the neutron dose estimations. Based on the resulted errors in neutron dose calculations by running a single input file for $2\;\times \;10^{9}$ particle histories, it was estimated that it should run an input file for a number of times and then the errors be combined from each run to have an acceptable error level. However, it was necessary to change the random seed number of the input file in each run to have a distinct run. In each input file, source particles were transported directly into a single run and the neutron dose was scored at various depths in the water phantom. For those programs which included prosthesis, each input file has been run 25 times (a total number of $5\;\times \;10^{10}$ source particle histories for each file). The program which did not include prosthesis was run 50 times (a total number of $10^{11}$ source particle histories). Energy cutoff for both electrons and photons were defined as 7 MeV in all input files. The neutron flux in the scoring cells was scored using F4 tally and then was converted to neutron equivalent dose rate (rem/h) using DE and DF cards. Neutron flux to equivalent dose rate conversion factors were extracted from Appendix H (Table H‐1) of MCNPX (version 2.6.0) manual.^(30)^ DIFs for total dose in the case of neutron contamination are presented as tabulated data. Herein the total dose was calculated as neutron dose plus photon dose. Photon dose needed for calculation of total dose was obtained utilizing F4 tally (flux) for photons. The F4 tally output values were then converted to photon doses (rem/h) using DE and DF cards extracted from Appendix H (Table H‐2) of MCNPX (version 2.6.0) manual.^(30)^ While the input files in calculation of photon dose were run for about $1.40\;\times \;10^{8}$ particles, the maximum Monte Carlo error in the tally cells was 1.36%. The method for calculation of combined uncertainties of DIF values for neutron contamination was the same as aforementioned for electron contamination.

In calculation of uncertainties in DIF values, uncertainty of dose at each point for the case presence of prosthesis was extracted from Monte Carlo output. The uncertainty of dose for the case of absence of prosthesis was also derived from the output. The combined uncertainties for division operation were calculated for various depths and prosthesis types and were reported in the tabulated data of DIF. Since the DIFs for total doses were obtained through division of total dose following presence of prosthesis to that in absence of prosthesis and the total dose is the summation of electron (neutron) dose by photon dose, the uncertainties related to the summation and division operations were combined toward calculation of uncertainties in DIF for total dose.

## III. RESULTS

### A. Percent depth dose and dose profiles

**Figure 2:**
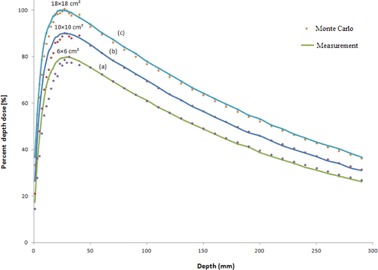
Percent depth dose values for 15 MV photon beam calculated by MC code versus measurement data (SSD = 100). The curves (a), (b) and (c) are corresponding to 6 × 6, 10 × 10, and $18\times 18\;{\text{cm}}^{2}$ field sizes, respectively. For more clarification, the data for 6 × 6 and $10\times 10\;{\text{cm}}^{2}$ field sizes are scaled 0.8 and 0.9, respectively.

**Figure 3:**
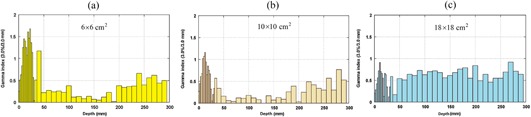
Gamma function results for PDD values for: (a) $6\times 6\;{\text{cm}}^{2}$, (b) $10\times 10\;{\text{cm}}^{2}$, and (c) $18\times 18\;{\text{cm}}^{2}$ field size. DD and DTA criteria were set as to 3% and 3 mm in the gamma calculations, respectively.

Percentage depth dose values which were obtained by MC simulations and measurements for 15 MV photon beam are plotted in Fig. 2. The data in the figure are related to 6 × 6, 10 × 10, and $18\times 18\;{\text{cm}}^{2}$ field sizes and SSD of 100 cm. As it was mentioned in the previous section, comparisons of the simulation and measurement data were based on gamma function calculations. The resulted gamma function values used for PDD comparisons for these three field sizes have been plotted in Fig. 3. Gamma calculations were performed using dose difference and distance to agreement criteria of 3% and 3 mm, respectively. As it can be seen from the gamma data in Fig. 3, only few points are having gamma values greater than unity, which is interpreted as fail or disagreement at these points. The points are related only to a few numbers of depths for 6 × 6 and $10\times 10\;{\text{cm}}^{2}$ field sizes. The number of points with gamma index of higher than unity were 11, 3, and 0 points for 6 × 6, 10 × 10, and $18\times 18\;{\text{cm}}^{2}$ field sizes, respectively. It should be noted that we have selected the calculation points very close to each other in the build‐up region. This resulted in a greater number of points with gamma index of higher than unity for the $6\times 6\;{\text{cm}}^{2}$ field.

**Figure 4:**
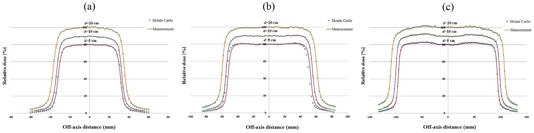
Dose profile data for 15 MV photon beam calculated by MC code versus measurement data (SSD = 100). The curves (a), (b), and (c) correspond to 6 × 6, 10 × 10, and $18\times 18\;{\text{cm}}^{2}$ field sizes, respectively. Dose profiles for 5, 10, and 20 cm depths are plotted in each part of (a), (b), and (c), respectively. For more clarification, the data for 5 and 10 cm depths are scaled 0.8 and 0.9 respectively.

**Figure 5:**
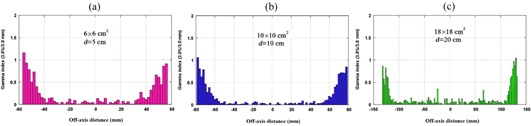
Gamma function results for dose profile data for: (a) $6\times 6\;{\text{cm}}^{2}$ field and depth of 5 cm, (b) $10\times 10\;{\text{cm}}^{2}$ field and depth of 10 cm, and (c) $18\times 18\;{\text{cm}}^{2}$ field and depth of 20 cm. DD and DTA criteria were defined as 3% and 3 mm in the gamma calculations, respectively.

Dose profiles related to the three field sizes of 6 × 6, 10 × 10, and $18\;\times \;18\;{\text{cm}}^{2}$ for three depths of 5, 10, and 20 mm, obtained from Monte Carlo simulations and measurements, are respectively plotted in Figs. 4(a)‐4(c), respectively. The corresponding gamma values for dose profile data for the three fields are plotted in Fig. 5. Figures 5(a), (b), and (c) illustrate gamma function results for dose profiles at 5, 10, and 20 cm depths, respectively. All the gamma calculations were performed by defining 3% dose difference and 3 mm distance to agreement criteria. From nine cases of gamma plots related to dose profiles (for three field sizes and three depths), we have only selected three sample cases to be plotted in Fig. 5. Among nine cases of dose profiles, the number of points with gamma index of higher than unity in each case was most frequently two points. The maximum number of points with gamma index of higher than unity was 5 points, which is related to depth of 20 cm for field of $10\times 10\;{\text{cm}}^{2}$. As it can be seen in Fig. 5, only gamma function values related to the penumbra regions in which there exist high‐dose gradients are greater than unity.

### B. Electron contamination

**Figure 6:**
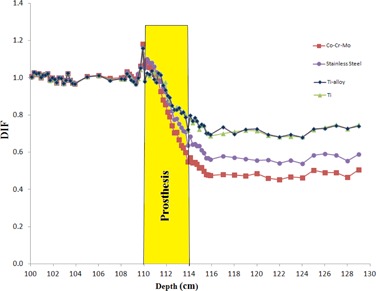
Dose increase factor (DIF) vs. depth for electron contamination in presence of hip prostheses in the 15 MV photon beam pathway for the four hip prostheses: Co‐Cr‐Mo, stainless steel, Ti, and Ti‐alloy.

Electron contamination in the form of dose increase factor (DIF) in the presence of hip prosthesis on the beam's central axis of Siemens PRIMUS 15 MV photon for the four prostheses as resulted from our MC simulations are shown in Fig. 6. This diagram indicates the ratio of electron contamination when the hip prosthesis was in the irradiation field to that without the hip prostheses (DIF) for various depths in a water phantom. In the calculations of DIF for electrons, electron contaminations were assessed for four common prostheses which are used in orthopedic procedures: Co‐Cr‐Mo, stainless steel, Ti‐alloy, and Ti. Each hip prosthesis was located at depth of 12 cm in the water phantom. As it is illustrated in the graph, the hip prosthesis (with dimensions of 4 × 4 × 4 cm) is located in the depth of 12 cm in the phantom. A numerical data of the DIF values for electron contamination are also listed in Table 2. DIF values related to total dose (electron dose + photon dose) for the four prostheses are presented in Table 3.

**Table 2:** Dose increase factor (DIF) assigned to electron contamination in a 15 MV photon beam produced by four prostheses: Co‐Cr‐Mo, Stainless steel, Ti‐alloy, and Ti in a water phantom

		*Dose Increase Factor (DIF) ± Uncertainty*			
	*Depth in Phantom (cm)*	*Co‐Cr‐Mo*	*Stainless Steel*	*Ti‐alloy*	*Ti*
	5	1.01±0.02	1.01±0.02	1.01±0.02	1.01±0.02
	6	1.01±0.02	1.01±0.02	1.01±0.02	1.04±0.02
	7	1.00±0.02	0.99±0.02	0.98±0.02	0.99±0.02
	8	1.00±0.02	0.99±0.02	0.99±0.02	1.00±0.02
	8.9	1.01±0.03	1.00±0.03	0.99±0.03	1.00±0.03
Water	9.1	0.99±0.03	0.99±0.03	0.98±0.03	0.98±0.03
	9.3	0.98±0.03	0.98±0.03	0.96±0.02	0.97±0.02
	9.5	1.02±0.03	1.02±0.03	1.00±0.03	1.00±0.03
	9.7	1.06±0.03	1.06±0.03	1.05±0.03	1.04±0.03
	9.9	1.18±0.03	1.16±0.03	1.16±0.03	1.14±0.03
	10.1	1.07±0.02	1.05±0.02	0.98±0.03	1.00±0.02
	10.3	1.09±0.02	1.10±0.03	1.03±0.03	1.02±0.02
	10.5	1.06±0.02	1.08±0.03	1.02±0.03	1.01±0.02
	10.7	1.05±0.02	1.08±0.03	1.04±0.03	1.03±0.02
	10.9	1.02±0.02	1.06±0.02	0.99±0.02	1.03±0.03
	11.1	0.98±0.02	1.03±0.02	0.97±0.02	0.98±0.02
	11.3	0.98±0.02	1.02±0.02	1.03±0.03	1.01±0.02
	11.5	0.93±0.02	0.98±0.02	1.01±0.03	1.02±0.03
	11.7	0.88±0.02	0.95±0.02	0.96±0.02	0.97±0.02
Prosthesis	11.9	0.86±0.02	0.92±0.02	0.93±0.02	0.98±0.02
	12.1	0.82±0.02	0.87±0.02	0.90±0.02	0.90±0.02
	12.3	0.79±0.02	0.86±0.02	0.89±0.02	0.88±0.02
	12.5	0.74±0.02	0.82±0.02	0.85±0.02	0.85±0.02
	12.7	0.70±0.02	0.78±0.02	0.83±0.02	0.83±0.02
	12.9	0.71±0.02	0.78±0.02	0.83±0.02	0.84±0.02
	13.1	0.67±0.02	0.75±0.02	0.84±0.02	0.84±0.02
	13.3	0.64±0.02	0.73±0.02	0.81±0.02	0.82±0.02
	13.5	0.62±0.02	0.71±0.02	0.81±0.02	0.82±0.02
	13.7	0.60±0.02	0.71±0.02	0.79±0.02	0.80±0.02
	13.9	0.55±0.01	0.64±0.02	0.72±0.02	0.72±0.02
	14.1	0.57±0.02	0.68±0.02	0.80±0.03	0.79±0.02
	14.9	0.52±0.02	0.64±0.02	0.74±0.02	0.72±0.02
	15.9	0.47±0.02	0.56±0.02	0.70±0.02	0.69±0.02
	17	0.48±0.02	0.58±0.02	0.73±0.02	0.70±0.02
Water	18	0.48±0.02	0.57±0.02	0.70±0.02	0.71±0.02
	19	0.47±0.02	0.56±0.02	0.72±0.02	0.72±0.02
	20	0.48±0.02	0.56±0.02	0.73±0.03	0.72±0.03
	21	0.46±0.02	0.56±0.02	0.69±0.03	0.69±0.02

**Table 3:** Dose increase factor (DIF) assigned to total dose (electron dose + photon dose) in a 15 MV photon beam produced by four prostheses: Co‐Cr‐Mo, Stainless steel, Ti‐alloy, and Ti in a water phantom

		*Dose Increase Factor (DIF) ± Uncertainty*			
	*Depth in Phantom (cm)*	*Co‐Cr‐Mo*	*Stainless Steel*	*Ti‐alloy*	*Ti*
	5	1.01±0.02	1.00±0.02	1.00±0.02	1.00±0.02
	6	1.01±0.02	1.01±0.02	1.01±0.02	1.01±0.02
	7	1.00±0.02	0.99±0.02	0.98±0.02	0.99±0.02
	8	1.00±0.02	0.99±0.02	0.99±0.02	0.99±0.02
	8.9	1.01±0.03	1.00±0.03	0.99±0.03	1.00±0.03
Water	9.1	0.99±0.03	0.99±0.02	0.98±0.02	0.98±0.02
	9.3	0.98±0.02	0.98±0.03	0.96±0.02	0.97±0.02
	9.5	1.02±0.03	1.02±0.03	1.00±0.03	1.00±0.03
	9.7	1.06±0.03	1.06±0.03	1.05±0.03	1.04±0.03
	9.9	1.18±0.03	1.16±0.03	1.16±0.03	1.14±0.03
	10.1	1.07±0.02	1.05±0.02	0.98±0.02	0.99±0.02
	10.3	1.09±0.02	1.10±0.03	1.02±0.02	1.02±0.02
	10.5	1.05±0.02	1.08±0.02	1.02±0.02	1.01±0.02
	10.7	1.05±0.02	1.08±0.02	1.04±0.02	1.03±0.02
	10.9	1.02±0.02	1.06±0.02	0.99±0.02	1.03±0.02
	11.1	0.98±0.02	1.03±0.02	0.97±0.02	0.98±0.02
	11.3	0.98±0.02	1.02±0.02	1.02±0.02	1.01±0.02
	11.5	0.93±0.02	0.98±0.02	1.01±0.03	1.02±0.03
	11.7	0.88±0.02	0.95±0.02	0.96±0.02	0.97±0.02
Prosthesis	11.9	0.85±0.02	0.92±0.02	0.93±0.02	0.97±0.02
	12.1	0.81±0.02	0.87±0.02	0.90±0.02	0.90±0.02
	12.3	0.79±0.02	0.86±0.02	0.89±0.02	0.88±0.02
	12.5	0.74±0.02	0.82±0.02	0.85±0.02	0.85±0.02
	12.7	0.70±0.02	0.77±0.02	0.83±0.02	0.83±0.02
	12.9	0.71±0.02	0.78±0.02	0.83±0.02	0.84±0.02
	13.1	0.67±0.02	0.75±0.02	0.84±0.02	0.83±0.02
	13.3	0.64±0.02	0.73±0.02	0.81±0.02	0.82±0.02
	13.5	0.62±0.02	0.71±0.02	0.81±0.02	0.82±0.02
	13.7	0.60±0.02	0.71±0.02	0.79±0.02	0.80±0.02
	13.9	0.55±0.01	0.64±0.02	0.72±0.02	0.72±0.02
	14.1	0.57±0.02	0.68±0.02	0.80±0.03	0.78±0.02
	14.9	0.52±0.02	0.63±0.02	0.74±0.02	0.72±0.02
	15.9	0.47±0.02	0.56±0.02	0.69±0.02	0.69±0.02
Water	17	0.48±0.02	0.58±0.02	0.73±0.02	0.70±0.02
	18	0.48±0.02	0.57±0.02	0.70±0.02	0.71±0.02
	19	0.47±0.02	0.56±0.02	0.72±0.02	0.72±0.02
	20	0.48±0.02	0.56±0.02	0.72±0.03	0.71±0.02
	21	0.46±0.02	0.56±0.02	0.69±0.02	0.69±0.02

### C. Neutron contamination

**Figure 7:**
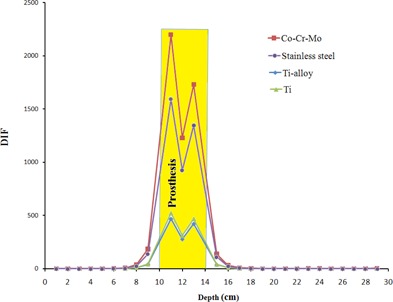
Dose increase factor (DIF) vs. depth for neutron contamination in presence of hip prostheses in the 15 MV photon beam pathway for the four hip prostheses: Co‐Cr‐Mo, Stainless steel, Ti‐alloy, and Ti.

Neutron contaminations in the form of DIF for neutrons in the presence of hip prosthesis on the central axis of beam are shown in Fig. 7. The data were obtained from MC simulations of the four hip prostheses when they are located at depth of 12 cm in a water phantom in a $10\times 10\;{\text{cm}}^{2}$ field of a 15 MV Siemens PRIMUS linac. This figure demonstrates the ratio of neutron dose with presence to that in the absence of hip prosthesis in the irradiation field (DIF) for the four prostheses materials. Dimensions of hip prosthesis in the simulation were 4 × 4 × 4 cm. Besides the graphical presentation of neutron contaminations in Fig. 7, numerical data of DIF values related to neutron productions from Co‐Cr‐Mo, stainless steel, Ti‐alloy, and Ti prostheses are listed in Table 4. DIF values corresponding to total dose (neutron dose + photon dose) for the four prostheses are listed in Table 5.

**Table 4:** Dose increase factor (DIF) assigned to neutron contamination in a 15 MV photon beam produced by four prostheses: Co‐Cr‐Mo, Stainless steel, Ti‐alloy, and Ti in a water phantom

		*Dose Increase Factor (DIF) ± Uncertainty*			
	*Depth in Phantom (cm)*	*Co‐Cr‐Mo*	*Stainless Steel*	*Ti‐alloy*	*Ti*
	1	1.05±0.11	1.02±0.10	1.04±0.10	1.06±0.10
	2	1.04±0.10	1.00±0.09	1.01±0.09	1.04±0.10
	3	1.03±0.10	0.97±0.10	0.96±0.09	1.01±0.10
	4	0.99±0.11	0.92±0.10	0.90±0.09	0.96±0.10
Water	5	1.05±0.12	0.93±0.11	0.84±0.10	0.98±0.11
	6	1.71±0.19	1.37±0.17	0.99±0.12	1.09±0.14
	7	5.85±0.61	4.58±0.55	1.90±0.23	2.17±0.27
	8	36.85±4.12	26.27±3.16	8.05±0.96	9.09±1.08
	9	184.55±21.80	137.33±18.75	40.66±4.93	43.17±5.20
	11	2197.63±298.29	1593.77±228.52	468.48±63.86	524.81±71.4
Prosthesis	12	1229.37±171.61	925.15±132.63	277.89±38.96	311.25±43.58
	13	1729.75±247.97	1347.23±105.44	422.54±40.84	471.48±67.80
	15	138.49±10.99	110.58±16.92	36.86±3.06	40.77±3.37
	16	31.05±4.79	23.49±3.62	8.49±1.36	9.49±1.51
	17	8.84±1.39	6.68±1.20	2.25±0.38	2.63±0.44
	18	2.86±0.53	2.17±0.42	0.93±0.19	1.15±0.23
Water	19	0.94±0.19	0.88±0.17	0.58±0.13	0.79±0.18
	20	0.39±0.08	0.71±0.16	0.60±0.12	0.49±0.11
	21	0.40±0.10	0.71±0.18	0.46±0.12	0.49±0.13
	22	0.83±0.26	0.45±0.13	0.49±0.15	0.46±0.15

**Table 5:** Dose increase factor (DIF) assigned to total dose (neutron dose + photon dose) in a 15 MV photon beam produced by four prostheses: Co‐Cr‐Mo, Stainless steel, Ti‐alloy, and Ti in a water phantom

		*Dose Increase Factor (DIF) ± Uncertainty*			
	*Depth in Phantom (cm)*	*Co‐Cr‐Mo*	*Stainless Steel*	*Ti‐alloy*	*Ti*
	1	1.00±0.08	1.00±0.08	1.00±0.08	1.00±0.08
	2	0.99±0.08	1.00±0.08	0.99±0.08	1.01±0.08
	3	1.01±0.10	0.99±0.09	1.00±0.09	0.99±0.09
	4	1.00±0.02	1.00±0.02	0.99±0.02	1.00±0.02
Water	5	1.00±0.02	1.00±0.02	1.00±0.02	1.00±0.02
	6	1.00±0.02	1.00±0.02	1.00±0.02	1.00±0.02
	7	1.00±0.02	1.00±0.02	1.00±0.02	1.00±0.02
	8	1.00±0.02	1.00±0.02	1.00±0.02	1.00±0.02
	9	1.00±0.02	1.00±0.02	1.01±0.02	1.00±0.02
	11	0.83±0.02	0.86±0.02	0.96±0.02	0.85±0.02
Prosthesis	12	0.68±0.01	0.75±0.02	0.88±0.02	0.79±0.02
	13	0.54±0.01	0.63±0.01	0.81±0.02	0.70±0.02
	15	0.44±0.01	0.55±0.01	0.69±0.02	0.68±0.02
	16	0.43±0.01	0.53±0.01	0.69±0.02	0.67±0.02
	17	0.43±0.01	0.52±0.01	0.68±0.02	0.67±0.02
	18	0.43±0.01	0.52±0.01	0.68±0.02	0.67±0.02
Water	19	0.43±0.01	0.52±0.01	0.68±0.02	0.66±0.02
	20	0.43±0.01	0.52±0.01	0.68±0.02	0.66±0.02
	21	0.43±0.01	0.52±0.01	0.67±0.02	0.66±0.02
	22	0.43±0.01	0.52±0.01	0.67±0.02	0.66±0.02

## IV. DISCUSSION

It is evident from Figs. 2 and 3 that there is a good agreement between our PDD values obtained by Monte Carlo simulations of linac head and the measured values. However, there are only a few points in gamma function data which have gamma values greater than unity which are related to high‐dose gradient parts in the build‐up region. For dose profiles, as plotted in Fig. 5, it can be noted that there are few points with gamma indexes greater than unity. These points are those located in the penumbra region or out of the radiation field. Generally speaking, there is a good agreement between our simulations and measurements of PDDs and dose profile. Therefore, our Monte Carlo model of the linac head is validated.

Evaluations of electron contaminations from the four hip prostheses (Fig. 6 and Table 2) are clearly indicating that electron contamination from Co‐Cr‐Mo prosthesis is higher than that of other prostheses. Maximum DIFs for electron contamination in the presence of prostheses are equal to 1.18, 1.16, 1.16 and 1.14 for Co‐Cr‐Mo, stainless steel, Ti, and Ti‐alloy, respectively. The dominant photon interaction in energies higher than 10 MeV is pair production and when a photon undergoes a pair production interaction, electron is produced. The probability of pair production is proportional to the square of atomic number $\left(Z^{2}\right)$. Atomic number of Co, Fe, and Ti (the most frequent elements in the Co‐Cr‐Mo, stainless steel, and Ti alloys) are 27, 26, and 22, respectively. Therefore, it can be expected that Co‐Cr‐Mo alloy would have the highest electron contamination production, followed by stainless steel, etc. When comparing DIF for electron contamination production by Ti‐alloy and Ti, the difference can be related to presence of V in Ti‐alloy with atomic number of 23, which does not exist in Ti prosthesis. Another effect that can be noticed from the data presented in Table 2 is the fact that electron contamination is increased before the hip prosthesis. This effect can be related to back‐scattered electrons from the prosthesis. This increase is seen up to a range of 3–5 mm away from hip prostheses. Electron contamination is reduced beyond prosthesis as a result of absorption of electrons in the prosthesis. The electron contamination is increased in some extent within the hip prostheses, but it has no significant effect since there is not any living tissue within the prostheses. The results of DIF for total dose (electron dose plus photon dose), which were presented in Table 3, indicate that the DIFs are showing the same trends as it was in the case of DIFs for electron contamination.

The effect of hip prostheses on neutron contamination on beam's central axis, as shown in Fig. 7 and Table 4, demonstrate that neutron contamination at the range of 5 cm before and 4 cm beyond the prostheses has been increased in the presence of prosthesis in the phantom. It is clearly shown in Fig. 7 that neutron contamination in presence of prosthesis is increased dramatically within the prosthesis. DIF values for neutron contaminations before prostheses amount to 184.55, 137.33, 40.66, and 43.17 for Co‐Cr‐Mo, stainless steel, Ti‐alloy, and Ti, respectively. This increase is higher for Co‐Cr‐Mo material, and then for stainless steel, Ti, and Ti‐alloy, respectively. It can be noticed from the data in Table 4 that unlike electron contamination, which is decreased beyond prosthesis and is increased only up to few millimeters before the prostheses, neutron contamination is increased both before and beyond the prosthesis. At it can be seen from the data of Table 5, DIF for total dose (neutron dose plus photon dose) is almost equal to 1.00 in the points above each four prostheses. The DIF shows decrease in both within prostheses and after them. It seems that the total dose is affected mainly by photon dose components and therefore it can be mentioned that, from total dose point of view, presence of prosthesis has not any effect on tissues before prosthesis, but it will attenuate the photons and thus the photon dose (and also total dose) after the prostheses is decreased. The decrease in photons dose inside the prostheses may not have any clinical aspect. There is not any previous study on total dose in presence on prostheses, but a previous study by Mesbahi and Nejad^(17)^ has shown similar trends in attenuation of photon dose with depth in presence of prostheses. There are some differences between the two studies, such as the photon energy (15 MV vs. 9 MV), linac model (Siemens PRIMUS vs. NEPTUN 10PC), etc.

## V. CONCLUSIONS

In the present study, we have simulated 15 MV photon beam of a Siemens PRIMUS linac by MCNPX MC code, and then electron and neutron dose increase effect due to electron and neutron contamination production from four hip prostheses was quantified. Our results have shown that presence of prosthesis in a phantom can increase both electron and neutron contaminations. Generally, the dose increase factor for neutron contamination is more than that for electron contamination. The neutron contamination, which originates from the prosthesis, can penetrate up to a range of 5 cm before the prosthesis and up to a range of 4 cm beyond it. Neutron contamination in the absence of hip prosthesis for Siemens PRIMUS linac is low, but it may be increased in the presence of prosthesis up to a factor of 184.55. The increase factor depends on the composition of prosthesis. When we consider the higher RBE of neutrons relative to photons, this effect can be more important with respect to biological damage to the patient.

It is evident from 2, 4 that Ti‐alloy and Ti prosthesis produce less electron and neutron contaminations than the other prostheses. Since a patient with hip junction replacement may undergo pelvic irradiation later in his lifetime, it is recommended to implant a Ti‐based alloy (Ti‐alloy or Ti) in his/her hip in his/her orthopedic procedure. Furthermore it is recommended to located prosthesis in the high‐energy photon beam pathway in the time of radiotherapy treatment planning. Based on the results acquired in this study, it is expected that, if a patient has any other type of prosthesis or metallic device implanted in his/her body (e.g., spinal fixation rods, dental restoration, fixed prosthodontics), if these devices are exposed to a high‐energy photon beam then there is a high possibility of electron and neutron contamination production. This field calls for further studies on the production of electron or neutron contaminations in high‐energy photon beams.

Presence of hip prosthesis can highly affect the total doses (electron dose plus photon dose; neutron dose plus photon dose). The total doses are reduced at depths after prosthesis in both cases of electron and neutron contaminations. This effect is due to attenuation of various particles inside the prosthesis, and is clinically important. Additionally the total dose in the case of electron contamination shows in depths increase before the prosthesis. This will increase the soft tissue dose which exists before hip bone. Taking into account these effects by clinical treatment planning systems may have influences on the treatment outcomes and, thus, it is recommended that the effects be considered in the process of treatment planning of a patient with hip prosthesis.

## ACKNOWLEDGMENTS

We are thankful to Dr. Faezeh Rahmani for her help in solving the neutron transport problems. The authors would also like to thank Reza Radiation Oncology Center (Mashhad, Iran) for providing the experimental data. The results of this study were extracted from the data of a research project under the grant number 900445, which was financially supported by Mashhad University of Medical Sciences (MUMS).
